# A Novel Method to Enable the Awareness Ability of Non-V2V-Equipped Vehicles in Vehicular Networks

**DOI:** 10.3390/s19092187

**Published:** 2019-05-11

**Authors:** Jian Wang, Qiang Zheng, Fang Mei, Weiwen Deng, Yuming Ge

**Affiliations:** 1 College of Computer Science and Technology, Jilin University, Changchun 130012, China; wangjian591@jlu.edu.cn (J.W.); zhengqiang16@jlu.edu.cn (Q.Z.); 2 Key Laboratory of Symbolic Computation and Knowledge Engineering of Ministry of Education, Jilin University, Changchun 130012, China; 3 School of Transportation Science and Engineering, Beihang University, Beijing 100191, China; wdeng@buaa.edu.cn; 4 Technology and Standards Research Institute, China Academy of Information and Communications Technology, Beijing 100191, China; geyuming@caict.ac.cn

**Keywords:** automated vehicles, V2V communications, non-V2V-equipped vehicle detection, license plate number recognition, calibration between camera and LiDAR, extension of traditional V2V systems

## Abstract

Autonomous vehicles need to have sufficient perception of the surrounding environment to produce appropriate driving behavior. The Vehicle-to-Vehicle (V2V) communication technology can exchange the speed, position, direction, and other information between autonomous vehicles to improve the sensing ability of the traditional on-board sensors. For example, V2V communication technology does not have a blind spot like a conventional on-board sensor, and V2V communication is not easily affected by weather conditions. However, it is almost impossible to make every vehicle a V2V-equipped vehicle in the real environment due to reasons such as policy and user choice. Low penetration of V2V-equipped vehicles greatly reduces the performance of the traditional V2V system. In this paper, however, we propose a novel method that can extend the awareness ability of the traditional V2V system without adding much extra investment. In the traditional V2V system, only a V2V-equipped vehicle can broadcast its own location information. However, the situation is somewhat different in our V2V system. Although non-V2V-equipped vehicles cannot broadcast their own location information, we can let V2V-equipped vehicle with radar and other sensors detect the location information of the surrounding non-V2V-equipped vehicles and then broadcast it out. Therefore, we think that a non-V2V-equipped vehicle can also broadcast its own location information. In this way, we greatly extend the awareness ability of the traditional V2V system. The proposed method is validated by real experiments and simulation experiments.

## 1. Introduction

Autonomous vehicles have modules such as environment sensing, behavioral decision making, path planning, and motion control [1], which can complete the driving task without the need for the driver. Therefore, they can replace human beings to complete some dangerous, messy, and boring driving tasks that humans are not willing to do. Autonomous vehicles are a key factor in achieving an intelligent transportation system and are widely regarded as a promising method to avoid road collisions and improve traffic conditions [2]. Autonomous vehicles have relied only on on-board sensors to collect information about the weather, road, and traffic conditions. Although the quality of these sensors has been greatly improved, they still have certain range and accuracy limitations [3].

The development of Intelligent Transport System (ITS) has led to the development of Vehicular Ad Hoc Networks (VANets) [4], which automatically creates a wireless network between vehicles to exchange messages. V2V technology allows connected vehicles to communicate with each other to get information about each other’s speed, position, and direction [5]. The messages that are broadcast by connected vehicles are called Cooperative Awareness Messages (CAM) and Decentralized Notification Messages (DENM). Therefore, through V2V technology, connected vehicles can communicate with any other connected vehicles in communication range. V2V technology extends the ability of autonomous vehicles using only on-board sensors. Autonomous vehicles’ systems can improve transportation system operations when they are combined with connected vehicles’ systems [6,7]. Improving the efficiency and reliability of operating autonomous vehicles and saving energy [8] are the main driving forces for the development of V2V technology.

**Figure 1:**
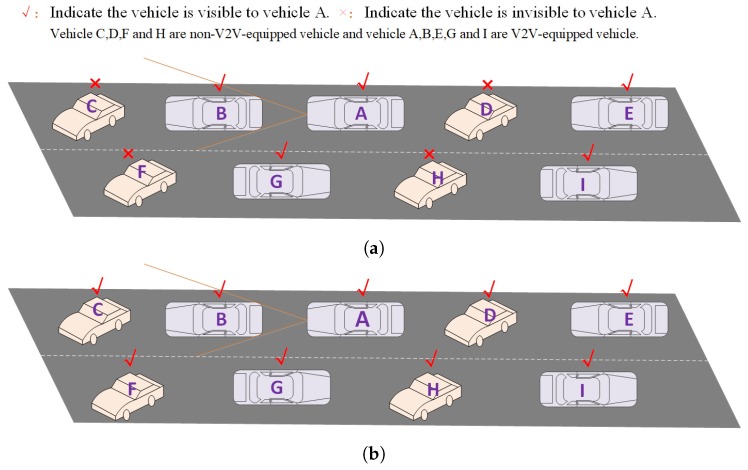
Our V2V system can extend the awareness ability of the traditional V2V system. (**a**) V2V-equipped Vehicle A cannot detect the non-V2V-equipped Vehicles C, D, F, and H in the traditional V2V system. (**b**) V2V-equipped Vehicle A can detect the non-V2V-equipped Vehicles C, D, F, and H in our V2V system.

However, due to the reasons of policy and user selection, the penetration of connected vehicles is very low in the actual environment, which greatly affects the performance of V2V systems; for example, in the following scenario (in Figure 1a): In the traditional V2V system, V2V-equipped Vehicle A cannot detect Vehicles C, D, F, and H, because the non-V2V-equipped vehicles (Vehicles C, D, F, and H) cannot broadcast their own location information. Given the present situation, this paper presents a novel idea of letting V2V-equipped vehicles detect location information and license plate numbers of the surrounding non-V2V-equipped vehicles and broadcast this information. Therefore, the non-V2V-equipped vehicles seem to have the ability to broadcast CAM as the V2V-equipped vehicles do. Consequently, in our V2V system, V2V-equipped Vehicle A can perceive Vehicles C, D, F, and H (see Figure 1b), because V2V-equipped Vehicle B can “tell” V2V-equipped Vehicle A where non-V2V-equipped Vehicle C is, V2V-equipped Vehicle E can “tell” V2V-equipped Vehicle A where non-V2V-equipped Vehicle D is, V2V-equipped Vehicle F can “tell” V2V-equipped Vehicle A where non-V2V-equipped Vehicle G is, and V2V-equipped Vehicle H can “tell” V2V-equipped Vehicle A where non-V2V-equipped Vehicle I is. In this way, we extend the awareness ability of non-V2V-equipped vehicles in vehicular networks under the low penetration of V2V-equipped vehicles.

The main focus of the present study is to investigate the awareness ability of connected vehicles and the whole V2V system under different market penetration rates of connected vehicles. In this paper, we used real experiments and simulation experiments; in addition, we designed different experimental scenarios. In each experimental scenario, we carried out experiments on different vehicle densities, different V2V-equipped vehicle penetration, and different detection rates to verify the reliability and feasibility of our method. The experimental results show that our method enables the awareness ability of non-V2V-equipped vehicles in vehicular networks under the low penetration of V2V-equipped vehicles.

The paper is structured as follows: in Section 2, an overview of related work is given. Afterwards, the subsequent Section 3 introduces the system architecture and algorithm implementation detail. Finally, Section 4 presents quantitative results from real-world test drives. The paper concludes with a summary in Section 5.

## 2. Related Work

Non-V2V-equipped vehicle detection is an important part of our V2V system. Non-V2V-equipped vehicle detection can be divided into laser data point clustering and non-V2V-equipped vehicle representation. Point clustering is classifying the collected points to make the properties of the points in the same cluster more similar. Non-V2V-equipped vehicle representation is a specific object representation problem. The representation model of a non-V2V-equipped vehicle is using some of its features, such as speed, position, and direction, during its tracking process.

### 2.1. Laser Data Point Clustering

The method of point clustering is to divide the scanning points into different clusters. There are some traditional point clustering methods, but these clustering methods do not achieve good clustering effects when applied to laser data. This is because the data points scanned by the LiDAR are distributed along the surface of the object being scanned, rather than concentrated around the center of the cluster. Therefore, the clustering method of laser scanning data cannot adopt the method based on the distance to the cluster center. Splitting the data into a known number of clustering methods is also not applicable to LiDAR scan data because the number of surrounding objects is not known when detecting dynamic environments.

Laser data point clustering can be divided into two methods based on Point Distance (PD) and the Kalman Filter (KF) [9]. Euclidean distance and Mahalanobis distance are the two most commonly-used distances in laser data clustering. The most common method is to compare the distance d($p_{i}$, $p_{j}$) between the two points ($p_{i}$ and $p_{j}$) and the distance threshold $d_{Th}$ [10,11]. When d($p_{i}$, $p_{j}$)< $d_{Th}$, point $p_{i}$ and point $p_{j}$ belong to the same cluster, otherwise they belong to different clusters. The distance threshold $d_{Th}$ can be defined in different ways; the easiest way is to set $d_{Th}$ to a constant value. In addition to this, there are other ways to determine the threshold adaptively. The distance threshold $d_{Th}=\frac{\rho_{i}-\rho_{j}}{\rho_{i}+\rho_{j}}$ was proposed in [12], which also considers the distance from the surface of the object to the sensor. In [13], another way to calculate the threshold was given: $d_{Th}=C_{0}+C_{1}\min\left(\rho_{i},\rho_{i+1}\right)$, where $C_{1}$ = $\sqrt{2(1-\cos\delta \theta )}$ = d($\rho_{i}$, $\rho_{i+1}$)/$\rho_{i}$ and $C_{0}$ is a constant value indicating radar noise. Compared with the previous method, it also considers the angular resolution parameter $C_{1}$. The algorithm in [14] was used to segment the raw data into meaningful portions and filter noise.

### 2.2. Object Representation

Object representation is an important part of our system, including point-based representations, feature-based representations, and rectangle-based representations. Examples based on point representation were presented in [15,16], which are suitable for tracking objects of a certainsize. In addition to the position of the object, we must also take the shape and size of the object into account. For obstacle detection, linear segments are the most common way of representing objects in indoor applications. The rectangle-based representation model is commonly used for tracking of dynamic objects. Since the surface of the vehicle is generally rectangular, the rectangular-based representation model not only exhibits the true size of the vehicle, but also has a very compact size. The rectangle-based representation model is used in our system, which defines a uniform standard for object representations of different shapes.

## 3. Method

In order to verify our idea, we developed a simple system that can obtain information (i.e., position, velocity, and time) of surrounding non-V2V-equipped vehicles and help the non-V2V-equipped vehicle to broadcast this information. In addition, we carried out simulation experiments to prove the feasibility of our method.

### 3.1. System Architecture

**Figure 2:**
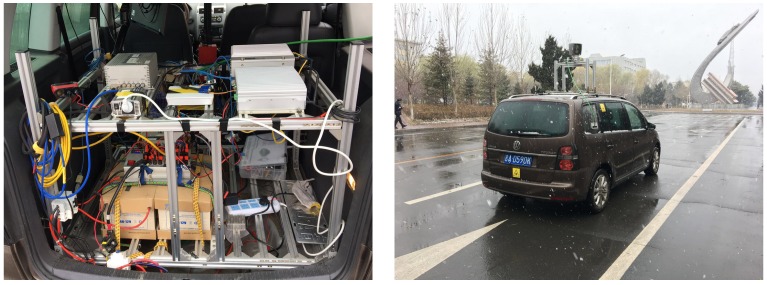
Experimental vehicle.

The “Where am I?” and “Where are they?” solutions aim to get the state of both the test vehicle and surrounding vehicles. The vehicle state information includes position, orientation, speed, and acceleration. Our test vehicle (see Figure 2) used on-board sensors (RT3002, LiDAR, and camera) to obtain the location information of surrounding non-V2V-equipped vehicles and capture license plate information.

#### 3.1.1. Introduction to System Hardware

**Figure 3:**
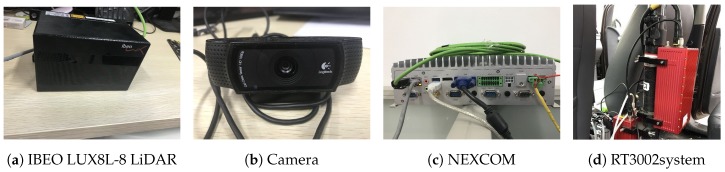
Hardware of the system.

The IBEO LUX8L-8 LiDAR sensor (Figure 3a) and Velodyne HDL-64E LiDAR sensor were used in our system to detect the information of surrounding objects. LiDAR sensor has high angular resolution and good mid-range detection ability. The scanning by laser pulses achieves a 3D representation of the environment suitable for the traffic scene. We can record the scanned data and use them with software tools to create models of the V2V-equipped vehicle’s surroundings.

Cameras were installed on the V2V-equipped vehicles to capture images of the surroundings. These images were then used for license plate number recognition. The cameras (in Figure 3b) we used were standard Logitech USB WebCam, and the price was only $30.

Our test vehicles were equipped with the RT3002 system to measure the vehicle’s position. The RT3002 system (in Figure 3d) mounted on our test vehicles is a precise inertial and GPS navigation system for measuring the motion, position, and direction of the object. It meets the test conditions of shock resistance and vibration resistance. The RT3002 system includes RT3002, a GPS system, a ground base station, a digital to analog conversion box, a mounting bracket, an antenna, an analog output cable, a CAN data transmission line, an RS232serial port cable, an Ethernet cable, an interface converter, and other accessories and cables. The RT3002 GPS system’s refresh frequency is 100 Hz; positioning accuracy: no ground base station 1.5 m; ground base station: 0.02 m. The ground base station has the following characteristics: all weather, waterproof, battery packaging, and signal transmission effective distance no less than 5 km. The RT3002 system’s output data are the position coordinates and heading angles in the world coordinate system and the three-axis components of the attitude angle, angular velocity, and velocity and acceleration in the body coordinate system (gyro coordinate system).

In order to process the data, we used an embedded PC named NEXCOM (in Figure 3c). Its CPU performance gives the users the ability to adapt to what they need in any telematics application. Its powerful graphic engine allows users to take full advantage of this product to achieve smooth, seamless, and stunning graphic performance on 3 different video outputs (VGA, DP, LVDS). In addition, the three SIM cards + dual WWAN modules architecture can increase the bandwidth for a faster data transfer speed.

#### 3.1.2. Coordinate System

**Figure 4:**
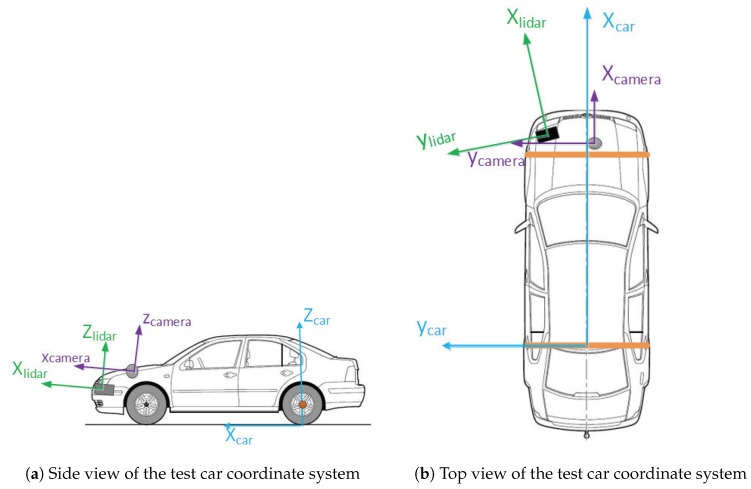
The coordinate system of the experimental car.

The coordinate system of our test vehicle is shown in Figure 4a,b, which represents the side view and the top view of the intelligent car coordinate system, respectively. The green coordinate system represents the IBEO LiDAR coordinate system $C_{LiDAR}(x_{LiDAR}$, $y_{LiDAR}$, $z_{LiDAR})$; the purple coordinate system represents the camera coordinate system $C_{camera}(x_{camera}$, $y_{camera}$, $z_{camera})$; and the blue coordinate system represents the coordinate system of the car $C_{car}(x_{car}$, $y_{car}$, $z_{car})$.

The information collected by IBEO and the camera is based on its own coordinate system. In order to unify the coordinate system, we adopted a coordinate calibration method, which will be discussed in Section 3.1.4. Finally, the IBEO coordinate and camera coordinate will be transformed into the car coordinate system.

#### 3.1.3. Vehicle Localization

In order to navigate successfully and safely, the vehicle must know information such as its position and orientation. This is called the “Where am I?” problem. In our test vehicles, we used RT3002 (see in Figure 3d) to obtain the position and direction of the car. The RT3000 product is a precise inertial and GPS navigation system for measuring the motion, position, and direction of the object. The system uses inertial navigation system technology and combines it with a GPS receiver with a high quality level. It coordinates the characteristics of the inertial navigation system and GPS to provide solutions.

#### 3.1.4. Calibration between LiDAR and Camera

Calibration between multiple sensors is a prerequisite in multi-sensor fusion-based applications. The result of the calibration highly influences subsequent fusion processes. A variety of methods have been developed to calibrate between camera and LiDAR. These calibration methods usually use a checkerboard and include a two-step process, intrinsic calibrations and extrinsic calibrations [17,18]. Two sources of error in the two-step calibration are likely to be produced.

In our system, however, we adopted a new calibration method between a camera and a 3D LiDAR using a triangle or diamond polygonal board [19]. The calibration method was to find a point-to-point correspondence between 2D images and 3D point clouds. The corresponding point pairs were used to solve the equation to obtain a calibration matrix for the equation. In this way, we only needed to find the projection matrix between the radar and the camera without estimating the two-step parameters. We can estimate the combined projection matrix of the extrinsic matrix and the intrinsic matrix without having to estimate them separately by this calibration method. The overall steps of the calibration method can be summarized as follows:

- Data acquisition: Place one or more triangle planar boards in front of the camera and 3D LiDAR. Take camera images, and measure the 3D point clouds of the 3D LiDAR for various locations of the board. To reduce the measured errors in the 3D LiDAR and to detect vertices of the triangle planar board in the image easily, it is recommended to use a bright monochromatic color for the board. Furthermore, the board color should be distinctive from the background, and the size of the board has to be large enough to include multiple laser scanning lines of the 3D LiDAR on the board surface.
- Matching 2D-3D point correspondences: Detect vertices of the triangle plane in images, and identify their corresponding 3D points from the laser scans by estimating the meeting points of two adjacent sides of the board.
- Estimate the calibration parameters between 3D LiDAR and camera. With the corresponding pairs, solve the linear equations for the initial estimate and refine the solutions for the final estimates.

### 3.2. Software Architecture

**Figure 5:**
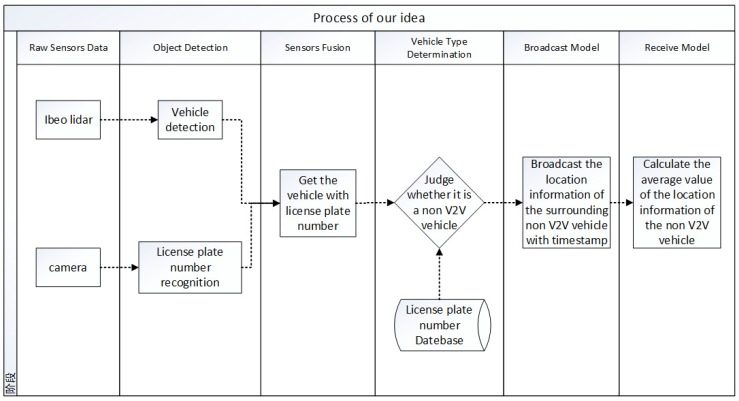
The process of our system.

The process of our system is shown as below (see Figure 5).

#### 3.2.1. Get Location Information of the Surrounding Vehicle

Getting the location of the surrounding non-V2V-equipped vehicles is an important part of our V2V system, which is the premise for the V2V-equipped vehicle to help the non-V2V-equipped vehicle to broadcast the message. The main task of this part is to process the LiDAR’s point cloud data to get the non-V2V-equipped vehicles’ relative position ($L_{relativeX}$, $L_{relativeY}$, $L_{relativeZ}$) and then obtain its own position ($L_{selfX}$, $L_{selfY}$, $L_{selfZ}$) according to the positioning system on the V2V-equipped vehicle, thereby obtaining non-V2V-equipped vehicles’ absolute position information ($L_{X}$, $L_{Y}$, $L_{Z}$), $L_{X}$ = $L_{selfX}$ + $L_{relativeX}$, $L_{Y}$ = $L_{selfY}$ + $L_{relativeY}$, $L_{Z}$ = $L_{selfZ}$ + $L_{relativeZ}$. The steps for obtaining the relative position information of the non-V2V-equipped vehicle are as follows.

- cluster LiDAR points, and remove the cluster whose point number is too small, so we get the cluster list.
- get the convex hull of each cluster.
- get the rectangle that represents the non-V2V-equipped vehicle.

**Figure 6:**
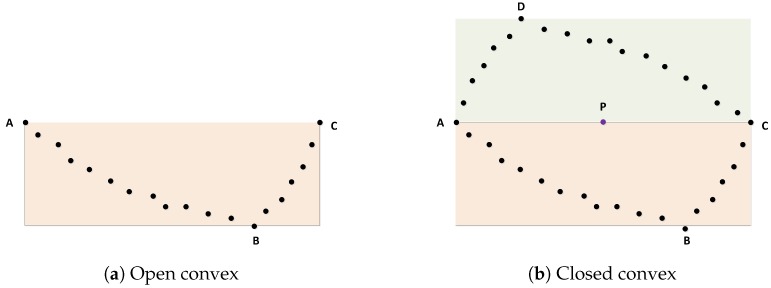
Open convex and closed convex.

There is a problem we should focus on, which is when we get the convex hull of a cluster. In fact, the convex contour is an open convex-hull, as shown in Figure 6a. This is due to the fact that only a part of a vehicle is visible. We connected the first and last points of the convex hull. However, this line cannot be considered as a vehicle edge because it represents the invisible part of the object. We assumed that the midpoint of this line is Pand that the invisible part of the vehicle and the outline of the visible part were symmetric about point P (see Figure 6b).

#### 3.2.2. Determine If a Vehicle Is a Non-V2V Vehicle

In the traditional V2V system, V2V-equipped vehicles do not consider whether a surrounding vehicle is a V2V-equipped vehicle. However, in our V2V system, it is necessary to judge whether the surrounding vehicles are non-V2V-equipped vehicle, which is the key to the implementation of our V2V system. At present, our V2V system uses the license plate number to judge. The specific judgment process is as follows. First of all, in our V2V system, every V2V-equipped vehicle will be registered, and information such as the license plate number will be entered into the database. When the V2V-equipped vehicles detect a nearby vehicle, our system uses the image captured by the camera mounted on the vehicle to identify the number of the license plate and obtain the number of the license plate. Then, we query in the database. If the license plate number is in the database, the surrounding vehicle is a V2V-equipped vehicle, otherwise, the surrounding vehicle is a non-V2V-equipped vehicle. It does not need to help the non-V2V-equipped vehicle broadcast the location information.

We used EasyPR, which is an open-source license plate recognition system in China to perform license plate number recognition. Compared with other license plate recognition systems, EasyPR has the following characteristics:

- It is based on the open source library openCV, which means that all of its code can be easily acquired.
- It can recognize Chinese.
- It has a higher recognition rate. At present, the character recognition can reach more than 90% accuracy.

**Figure 7:**
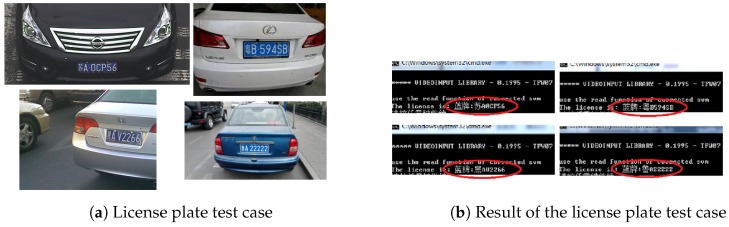
License recognition.

As for license plate number recognition, you can learn more in [20,21,22,23]. Figure 7 shows the license recognition model of our V2V system. The left of Figure 7a is the test cases, and the right of Figure 7b shows the test result of the left test cases.

Delegating safety message dissemination tasks to another vehicle will lead to serious security issues in V2X communications. For example, direct identification of vehicles such as the plate number should not be used to identify the other vehicles. We used Algorithms 1 and 2 from [24] to avoid this privacy exposure issue.

**Algorithm 1:** Encryption protocol performed by $V_{b}$**Input**: Pseudonym $PN_{\left(a,i\right)}^{j}$ of $V_{b}$ and message *M*. 1: Verify $SIG(\tau_{\left(a,i\right)}^{j},t_{\left(a,i\right)},S_{R_{i}})$, and compute $\lambda_{\left(a,i\right)}^{j}=e\left(\tau_{\left(a,i\right)}^{j},\sigma_{a}^{j}P\right)$ 2: Choose $k\in {\left\{0,1\right\}}^{n}$ randomly. 3: Compute $\rho =H_{2}\left(k,M\right)$ 4: Compute cipher text as $C=\langle H\left(\rho P\right)⊕{\left(\lambda_{\left(a,i\right)}^{j}\right)}^{k},e{\left(P,\sigma_{a}^{j}|P\right)}^{k},M⊕H_{1}\left(e\left(\sigma_{a}^{j}P,H\left(\rho P\right)P\right)\right)\rangle$ 5: Transmit *C* to $V_{a}$.

Algorithm 1 expounds the encryption protocol used by $V_{b}$ to send a message to $V_{a}$, where $\tau_{\left(a,i\right)}^{j}$ is the pseudonym, $t_{\left(a,i\right)}$ is the pseudonym expiration time, $S_{R_{i}}$ is the private key, and $SIG(\tau_{\left(a,i\right)}^{j},t_{\left(a,i\right)},S_{R_{i}})$ is the signature to ensure that $V_{a}$ is a genuine member of the system and has been authenticated by an RSU. For verification, $V_{b}$ first verifies $Cert_{R}$ using $P_{MD}$ and then uses $R_{i}$ from $Cert_{R}$ to verify signature $SIG(\tau_{\left(a,i\right)}^{j},t_{\left(a,i\right)},S_{R_{i}})$. If verification is successful, $V_{b}$ computes $\lambda_{\left(a,i\right)}^{j}=e\left(\tau_{\left(a,i\right)}^{j},\sigma_{a}^{j}P\right)$. To encrypt the plain-text message $M\in {\left\{0,1\right\}}^{n}$ for $V_{a}$ with pseudonym $PN_{\left(a,i\right)}^{j}$, $V_{a}$ performs Steps 2–4 of Algorithm 1. Symbol ⊕ stands for the XOR operation.

**Algorithm 2:** Decryption protocol performed by $V_{a}$**Input**: $C=\langle U,V,W\rangle ,S_{\left(a,i\right)}^{j}$ 1: Compute $\Gamma_{\left(a,i\right)}^{j}=U⊕V^{S_{\left(a,i\right)}^{j}}$ 2: Retrieve $M=W⊕H_{1}\left(e\left(\sigma_{a}^{j}P,\Gamma_{\left(a,i\right)}^{j}P\right)\right)$

Algorithm 2 expounds the decryption protocol used by $V_{a}$ to decrypt ciphertext *C* sent by $V_{b}$. We denote ciphertext *C* using the tuple C=〈U,V,W〉, where $U=H\left(\rho P\right)⊕{\left(\lambda_{\left(a,i\right)}^{j}\right)}^{k}$, $V=e{\left(P,\sigma_{a}^{j}|P\right)}^{k}$, and $W=M⊕H_{1}\left(e\left(\sigma_{a}^{j}P,H\left(\rho P\right)P\right)\right)$. The protocol is fairly self-explanatory. The decryption of it is performed using private key $S_{\left(a,i\right)}^{j}$.

#### 3.2.3. The Strategy of Receiving Safety Messages

Assume a scenario where there is more than one V2V-equipped vehicle around a non-V2V-equipped car. Therefore, this non-V2V-equipped vehicle is detected by multiple V2V-equipped vehicles at the same time, and these nearby V2V-equipped vehicles broadcast the location information (i.e., position, velocity, and time) of this non-V2V-equipped vehicle simultaneously. The other V2V-equipped vehicles in the LANmay receive multiple packets of information about the same non-V2V-equipped car simultaneously. Therefore, how does the other car choose these data packets? Does the other car choose one or all of these packages? In order to avoid accidental errors caused by receiving only one packet, we adopt the strategy of receiving all these packets. Then, how do we deal with these packets? The easiest way is to calculate the average of the data in these packets. The V2V-equipped vehicles that broadcast data packets of this non-V2V-equipped vehicle are marked as $v_{1},v_{2},...,v_{n}$, and the broadcast data packets are respectively recorded as $P_{v1},P_{v2},...,P_{vn}$, while the corresponding position information in the data packet is respectively marked as $L_{v1},L_{v2},...,L_{vn}$, the speed information as $V_{v1},V_{v2},...,V_{vn}$, the time information as $t_{1},t_{2},...,t_{n}$, and the timestamp of the packets as $t_{p1},t_{p2},...,t_{pn}$. The time of a V2V-equipped vehicle in a local area network receiving multiple packets of a non-V2V-equipped vehicle is marked as $T_{recv1},T_{recv2},...,T_{recvm}$. When a V2V-equipped vehicles in the LAN received multiple packets of the same non-V2V-equipped vehicles, we used the following calculation strategy: When this V2V-equipped vehicle first receives the packet about this non-V2V-equipped vehicle, the time of receiving this packet is marked as $T_{first}$, and the timestamp in the packet (the timestamp in the packet represents the time that the V2V-equipped vehicle detects the non-V2V-equipped vehicle) is marked as first. We do not need to do any calculation at this time, just put the packet into a packet queue. Then, when the data packet broadcast by other V2V-equipped vehicles of this non-V2V-equipped vehicle is received, the data packet is added to the above queue as long as the receiving time $T_{recvi}$ and the timestamp $t_{i}$ in the data packet satisfy the following two conditions.

(1) $$\left\{\begin{array}{cc} T_{first}⩽T_{recvi}⩽T_{first}+T_{th}\\ |t_{first}-t_{i}|⩽t_{th} & 1⩽i⩽m \end{array}\right.$$

The value mrepresents the size of the queue, that is the number of packets in the queue. $T_{th}$ is a time threshold. Finally, we calculate the average position ($\bar{L}$), average speed ($\bar{V}$), and other information of the non-V2V-equipped vehicle at the time point $t_{x}$ according to the data packets in the queue.

(2) $$\left\{\begin{array}{c} \bar{t_{x}}=\frac{\sum_{i=1}^{m}t_{m}}{m}\\ \bar{L}=\frac{\sum_{i=1}^{m}L_{vm}}{m}\\ \bar{V}=\frac{\sum_{i=1}^{m}V_{vm}}{m} \end{array}\right.$$

## 4. Experiments and Simulation Results

We will conduct actual experiments and simulation experiments to verify the reliability and feasibility of our method, respectively. The traditional V2V system means that all V2V-equipped vehicles in the system will only broadcast their own location information, while our V2V system means that the V2V-equipped vehicles in the system will help the nearby non-V2V-equipped vehicles broadcast location and other information.

### 4.1. Method Feasibility Simulation

**Figure 8:**
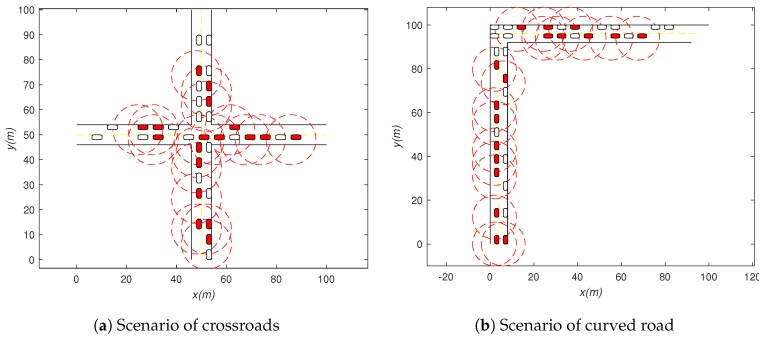
Scenario of method feasibility simulation.

We will validate the feasibility of our method in the crossroads scenario (Figure 8a) and bend scenario (Figure 8b), respectively. A Java simulation platform was developed to simulate road scenarios and vehicle models and generate relevant data, and then, MATLAB was used to render data. The red rounded rectangle and white rounded rectangle represent the V2V-equipped vehicle and non-V2V-equipped vehicle, respectively. The red dotted circle represents the visual detection range of the V2V-equipped vehicles. Table 1 gives some parameters of the V2V system.

**Figure 9:**
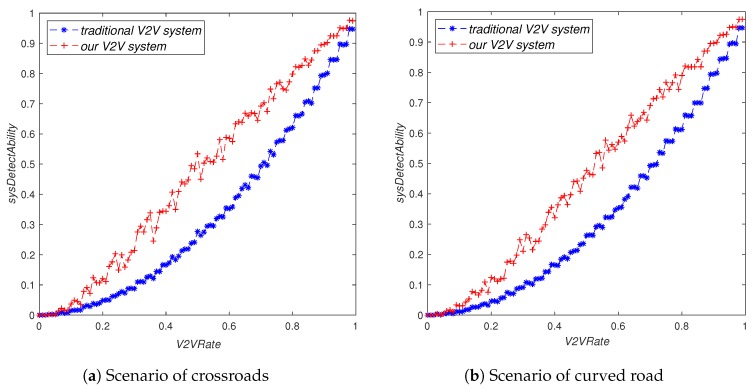
The influence of V2VRate on sysDetectAbility.

As can be seen from Figure 9, whether in the traditional V2V system or our V2V system, the system’s perception ability sysDetectAbility will increase with the increase of V2VRate. However, our V2V system was obviously better than the traditional V2V system, especially when V2VRate was in the interval [0.3, 0.7].

**Figure 10:**
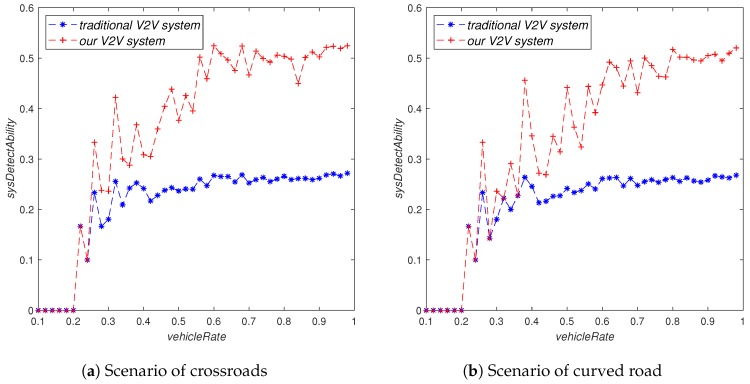
The influence of vehicleRate on sysDetectAbility.

As can be seen from Figure 10, vehicle density did not have a significant impact on the system’s perception ability in the traditional V2V system. However, in our V2V system, the system’s perception ability will increase significantly with the increase of vehicle density. Moreover, our V2V system was obviously better than the traditional V2V system under the same vehicle density. It can also be seen from Figure 10 that when the vehicle density was very high, especially when it was greater than 0.5, the increase of vehicle density had less and less influence on the system’s perception ability.

**Figure 11:**
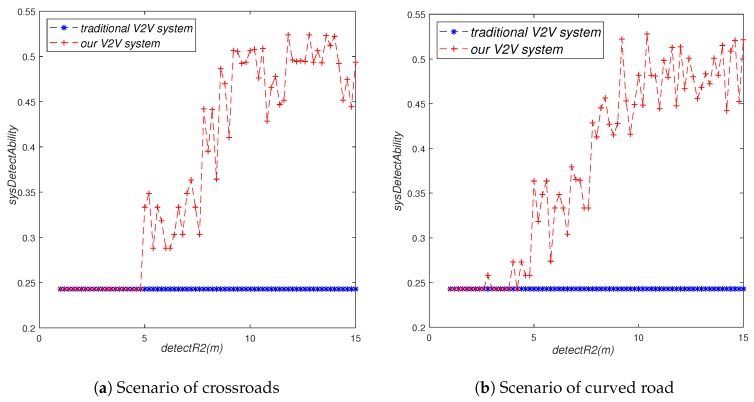
The influence of detectR2 on sysDetectAbility.

As can be seen from Figure 11, visual detection range did not have any impact on the system’s perception ability in the traditional V2V system. However, in our V2V system, the system’s perception ability will increase significantly with the increase of visual detection range. Moreover, our V2V system was significantly better than the traditional V2V system under the same visual detection range. As can be seen from the figure, the system’s perception ability did not increase significantly with the increase of visual detection range when the visual detection range was small (it is less than five meters in our figure). This is because when the visual detection range is small, there will not be many vehicles in the visual detection range of V2V-equipped vehicles, let alone many non-V2V-equipped vehicles. When the visual detection range was of a medium size (it is larger than five meters and less than 10 m in our figure), the system’s perception ability increased significantly with the increase of visual detection range; when the visual detection range exceeded a certain size (it is larger than 10 m in our figure), the system’s perception ability did not change significantly with the increase of visual detection range.

In addition, we also present the results in the packet delivery ratio, delay, and throughput.

**Figure 12:**
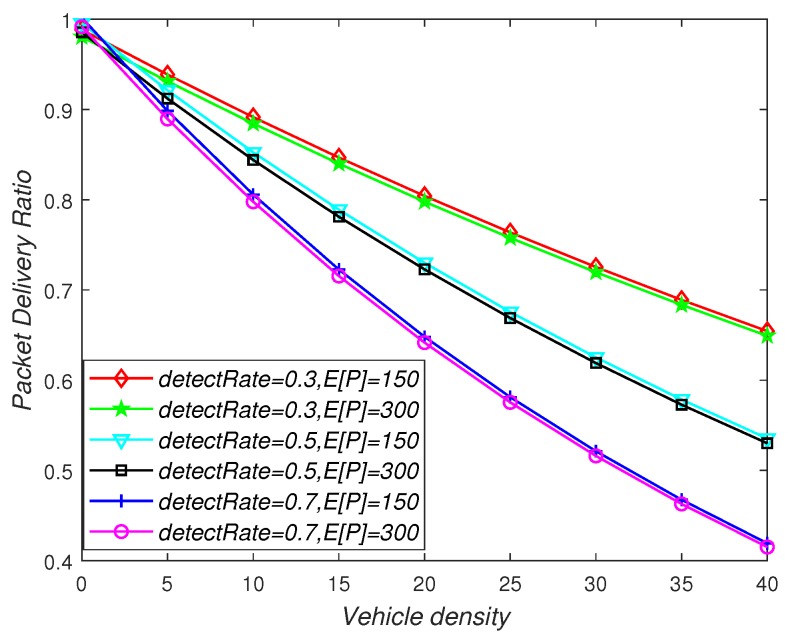
The influence of vehicle density, detectRate, and packet payload size on the packet delivery ratio.

Figure 12 depicts the effects of vehicle density, detectRate, and packet length on the Packet Delivery Ratio (PDR). As can be seen from the figure, PDR decreased as the vehicle density increased, because when the vehicle density increased, the error rate of the data packet became large, and the data packet that cannot be received in time became an invalid data packet. The increase of detectRate will cause the V2V-equipped vehicles in the V2V system to help the non-V2V-equipped vehicles to transmit more data packets, causing the channel to be busy, increasing the probability of the node losing the packet, and PDR decreasing. In addition, the longer the length of the data packet, the higher the arrival rate, and the smaller the PDR, because in this case, the service time of the channel is too long, causing the channel to be busy, resulting in packet transmission failure.

**Figure 13:**
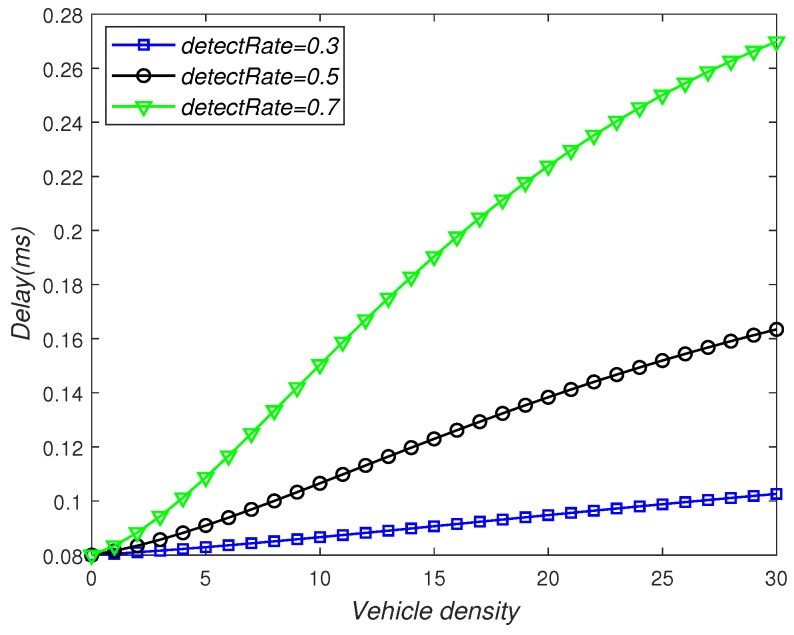
The influence of vehicle density and detectRate on delay.

Figure 13 depicts the effect of detectRate and vehicle density on delay. As can be seen from the figure, the delay time increased as the vehicle density increased, as each V2V-equipped vehicle needed to compete with more neighbors for channel access. At the same time, the delay time increased as the detectRate increased. Because the V2V-equipped vehicles needed to help the non-V2V-equipped vehicles to deliver more data packets, resulting in a busy channel and a large delay.

**Figure 14:**
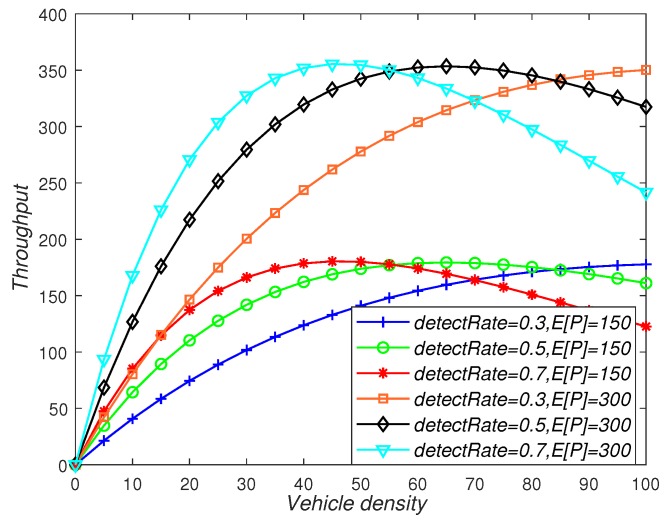
The influence of vehicle density, detectRate, and packet payload size on throughput.

Figure 14 depicts the effects of vehicle density, detectRate, and packet length on throughput. As can be seen from the figure, as the vehicle density increased, the trend of throughput increased first and then decreased. In addition, the detectRate determined the drop point of throughput. For example, when detectRate = 0.3, the throughput decreased when the vehicle density was 45. In addition, the throughput increased as the length of the packet increased, so appropriately increasing the length of the packet can improve the reliability of vehicle communication.

### 4.2. Method Reliability Experiment

We conducted a Forward Collision Warning (FCW) experiment and Collision Warning At Crossroads (CWAC) experiment separately to test the reliability of our system.

#### 4.2.1. Forward Collision Warning Experiment

**Figure 15:**
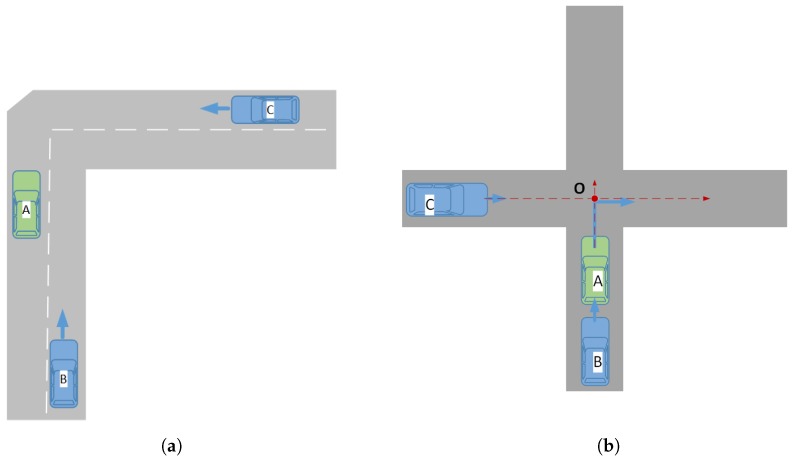
Scenario of our experiment. (**a**) Scenario of the Forward Collision Warning (FCW) experiment. (**b**) Scenario of the Collision Warning At Crossroads (CWAC) experiment.

The experiment scenario is shown in Figure 15a. There was a sharp turning road with three vehicles on it. Vehicles B and C were V2V-equipped vehicles, and Vehicle A was a non-V2V-equipped vehicle. Vehicle C moved from the right to the left, and Vehicle B moved from the bottom to the top. For some reason, Vehicle A temporarily stopped on the edge of the road. Vehicle A was in the perception range of Vehicle B, but not in the perception range of Vehicle C due to the angle or other reasons. Moreover, Vehicle A was a non-V2V-equipped vehicle, which could not broadcast its own location information, so Vehicle C could not perceive the existence of Vehicle A in time, which would undoubtedly have a potential collision risk. However, in our system, Vehicle B detected the location information of Vehicle A and broadcast it. Therefore, it seems that the Vehicle A was broadcasting information on its own. Vehicle C can have a corresponding reaction by the location information of Vehicle A broadcast by Vehicle B to avoid potential collision risk.

**Figure 16:**
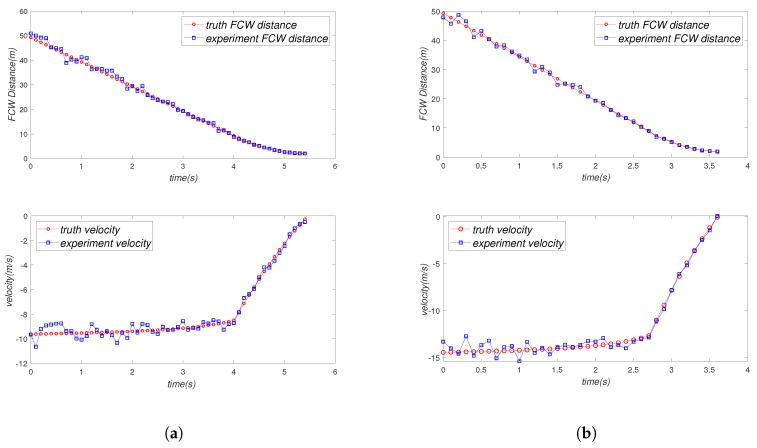
Result of FCW Experiment 1. (**a**) Initial FCW distance is 50 m, and initial velocity is 10 m/s. (**b**) Initial FCW distance is 50 m, and initial velocity is 15 m/s.

**Figure 17:**
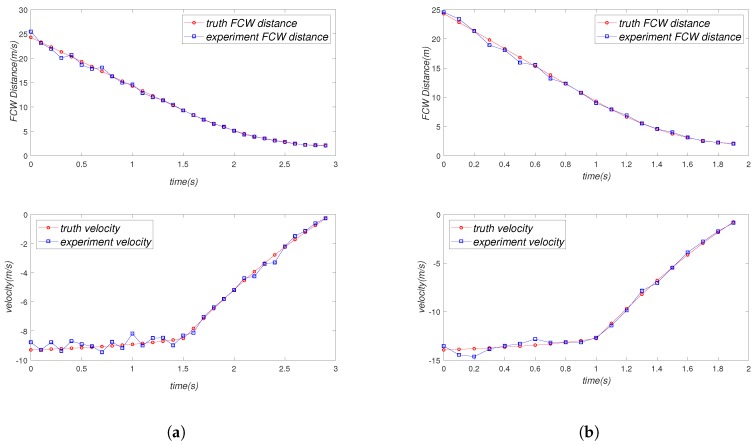
Result of FCW Experiment 2. (**a**) Initial FCW distance is 25 m, and initial velocity is 10 m/s. (**b**) Initial FCW distance is 25 m, and initial velocity is 15 m/s.

In order to simplify the experiment, we made Vehicle C start with a uniform linear motion. Vehicle C should perform a linear motion of deceleration when it is 10 m away from Vehicle A, and the final braking position of Vehicle C should be two meters away from Vehicle A. In our experiments, we gave Vehicle C different initial speeds and different initial positions to validate the reliability of our experiments. The corresponding experimental results are shown in Figure 16 and Figure 17. We drew the curve of real FCW distance and velocity and experimental FCW distance and velocity, respectively, and compared the fitting degree of the curves as the basis of the reliability of our experiment.

Table 2 is the sampling data of our FCW experiment. We sampled every 0.5 s and recorded the truth location (position and velocity) and the experiment location. Then, we calculated the average error of two groups of experiments respectively. It can be seen in the table that the error rate was very small (2.65% and 4.95%), which means that our system was very reliable in the forward collision warning scenario.

#### 4.2.2. Collision Warning at Crossroads Experiment

The CWAC experiment scenario is shown in Figure 15b. There were three vehicles (A, B, C) driving at a crossroads. Vehicles B and C were V2V-equipped vehicles, and Vehicle A was a non-V2V-equipped vehicle. Vehicle C was driving from left to right, and Vehicle B and Vehicle A were driving from bottom to up. We assumed that Vehicle A turned right at the intersection and Vehicle C kept moving from left to right. It is easy to see that Vehicle C and Vehicle A will collide at point O. We can find out the coordinates of the collision point O according to the direction information of Vehicle A and Vehicle C. Then, we calculated the time t1 and time t2 required for A and C to reach point O in real time. The time difference *t* (t=t1−t2) was calculated as a standard to measure the level of collision risk. We drew the curve of the real t and experiment t, respectively, and compared the fitting degree of the two curves as the basis of the reliability of our experiment.

**Figure 18:**
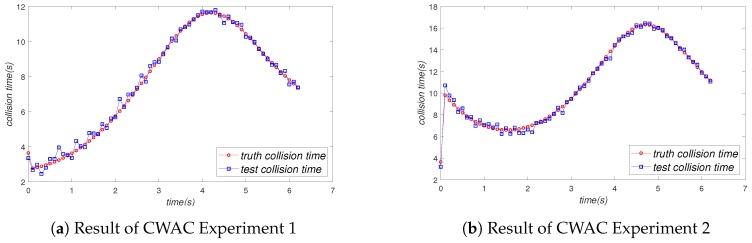
Result of the CWAC experiment.

In order to simplify the experiment, we let Vehicle A and Vehicle B maintain uniform linear motion and Vehicle C perform variable speed straight line motion. In the first group of experiments, the speed trend of Vehicle C was a sine function. In the second group of experiments, the speed trend of Vehicle C was a cosine function. The experiment result of the first group of experiments and the second group of experiments is shown in Figure 18a,b, respectively. The red curve and blue curve in Figure 18a,b show the real collision time and the experimental collision time, respectively. As we can see from Figure 18a,b, the red curve and blue curve had a very high fitting degree, which means our system is very reliable.

Table 3 is the sampling data and truth data of our experiments. The truth data were the theoretical value of the formula calculation, that is the speed of the vehicles followed a mathematical function. We can calculate the truth data (truth velocity and truth position) by mathematical function and integration. We sampled every 0.8s and recorded the truth collision time and the experimental collision time, respectively. Then, we calculated the average error rate of experiments. It can be seen from Table 3 that the error rate of the experiments was very small (1.49% and 5.28%), which means that our system is very reliable in the CWAC scenario.

## 5. Conclusions and Future Work

In this paper, we proposed a novel method to solve the problem of low penetration of V2V-equipped vehicles (connected vehicles) and improve the awareness ability of the traditional V2V system. In the traditional V2V system, it is difficult to ensure that all vehicles are V2V-equipped vehicles in the real environment, which greatly reduces the reliability of the entire V2V system. V2V-equipped vehicles only periodically broadcast their own location information (location, speed, etc.) in the traditional V2V system. In view of this, we proposed a novel method for expending the traditional V2V system. The core of our idea was that the V2V-equipped vehicles help the surrounding non-V2V-equipped vehicles broadcast location information. Therefore, it seems that non-V2V-equipped vehicles have the ability to broadcast their own location information, which greatly expands the awareness ability of the traditional V2V system. In the experimental part, we verified the feasibility and reliability of our method. We will do further research on the performance of license plate recognition to improve the reliability of our system.

## Figures and Tables

**Table 1:** Parameters introduced into the V2V system.

Parameters	Meaning
$V_{i}$	V2V-equipped vehicles
$detectR_{2}$	visual detection radius of $V_{i}$
$R_{1}$	communicate radius of $V_{i}$
totalV2VVehicles	the number of total V2V-equipped vehicles in the V2V system
$n_{i}$	the number of vehicles in the visual detection radius ($detectR_{2}$) of $V_{i}$
$vn_{i}$	the number of V2V-equipped vehicles in the visual detection radius ($detectR_{2}$) of $V_{i}$
$nn_{i}$	the number of non-V2V-equipped vehicles in the visual detection radius ($detectR_{2}$) of $V_{i}$
$cannn_{i}$	the number of non-V2V-equipped vehicles that can be detected by the on-board sensors of $V_{i}$
detectRate	detection ratio of $V_{i}$, detectRate= $\frac{cannn_{i}}{nn_{i}}$
$N_{i}$	the number of vehicles in the communicate radius of $V_{i}$
$VN_{i}$	the number of V2V-equipped vehicles in the communicate radius ($R_{1}$) of $V_{i}$
$NN_{i}$	the number of non-V2V-equipped vehicles in the communicate radius ($R_{1}$) of $V_{i}$
$CANNN_{i}$	the number of non-V2V-equipped vehicles that other V2V-equipped vehicles “tell” $V_{i}$ about (excluding $cannn_{i}$)
$detectAbility_{i}$	the perception ability of $V_{i}$, in the traditional V2V system, dectectAbility = $\frac{VN_{i}}{N_{i}}$, in our V2V system, detectAbility = $\frac{VN_{i}+CANNN_{i}}{N_{i}}$
sysDetectAbility	the perception ability of the whole V2V system, $sysDetectAbility=\sum_{i=1}^{totalV2VVehicles}detectAbility_{i}$
V2VRate	the proportion of V2V-equipped vehicles in the V2V system
vehicleRate	vehicle density of the V2V system

**Table 2:** Data samples of the FCW experiment.

Sampling Time (s)	Truth Position (m)	Test Position (m)	Truth Velocity (m/s)	Test Velocity (m/s)
0.1	42.8	41.5054	−14.3604	−14.4186
0.6	35.3000	34.4359	−14.2387	−14.7245
1.1	27.8000	26.6357	−14.0598	−13.9693
1.6	20.3000	19.9314	−13.7710	−13.9774
2.1	12.8000	12.9106	−13.2265	−13.6934
2.6	5.9490	5.7122	−8.5547	−8.1722
3.1	2.4154	2.4615	−2.4063	−2.3768
3.2	2.1413	2.1830	−1.3771	−1.5846
3.3	2.0115	2.0859	−0.3896	−0.3336
average error		2.65%		4.95%
max error		4.85%		24%

**Table 3:** Data samples of the CWAC experiment.

Sampling Time (s)	Truth Collision Time 1 (s)	Test Collision Time 1 (s)	Truth Collision Time 2 (s)	Test Collision Time 2 (s)
0.1	9.8096	10.2855	2.7760	2.0849
0.9	7.1614	7.0127	3.4905	3.6553
1.7	6.6381	6.6739	4.9770	5.4012
2.5	7.8600	7.5681	7.2779	6.5011
3.3	10.8155	10.7973	10.0125	10.0022
4.1	14.8072	14.8008	11.6181	11.4639
4.9	16.1760	16.2989	10.6826	10.8824
5.7	13.2747	12.9067	8.5324	8.5928
average error		1.49%		5.28%
max error		4.85%		23.8%
